# Identification and imaging of miR-155 in the early screening of lung cancer by targeted delivery of octreotide-conjugated chitosan-molecular beacon nanoparticles

**DOI:** 10.1080/10717544.2018.1516003

**Published:** 2019-01-09

**Authors:** Hai-Zhen Zhu, Jing Hou, Yi Guo, Xin Liu, Fei-Long Jiang, Guang-Peng Chen, Xiu-Feng Pang, Jian-Guo Sun, Zheng-Tang Chen

**Affiliations:** a Department of Oncology, Guizhou provincial people's Hospital, Guizhou, China;; b Department of Breast surgery, Guizhou provincial people's Hospital, Guizhou, China;; c Department of Basic knowledge, Guiyang nursing vocational college, Guizhou, China;; d Department of Clinical laboratory, Guizhou provincial people's Hospital, Guizhou, China;; e Department of Oncology, Chinese Medicine Hospital of Chongqing, Chongqing, China;; f Cancer Institute of PLA, Xinqiao Hospital, Army Medical University, Chongqing, China;; g Shanghai Key Laboratory of Regulatory Biology, Institute of Biomedical Sciences and School of Life Sciences, East China Normal University, Shanghai, China

**Keywords:** Lung cancer；microRNA-155；molecular beacon；chitosan nanoparticles；molecular imaging

## Abstract

Lung cancer is still the most common cancer globally. Early screening remains the key to improve the prognosis of patients. There is currently a lack of specific and sensitive methods for early screening of lung cancer. In recent years, studies have found that microRNA plays an important role in the occurrence and development of lung cancer and become a biological target in the early diagnosis of lung cancer. In this study, lung cancer cells, subcutaneous xenografts of lung cancer in nude mice, and Lox-Stop-lox K-ras G12D transgenic mice were used as models. The transgenic mice displayed the dynamic processes from normal lung tissue to atypical hyperplasia, adenomas, carcinoma *in situ* and lung adenocarcinoma. It was found that miR-155 and somatostatin receptor 2 (SSTR2) were expressed in all the disease stages of transgenic mice. Through molecular beacon (MB) technology and nanotechnology, chitosan-molecular beacon (CS-MB) nanoparticles and targeted octreotide (OCT) were conjugated and synthesized. The octreotide-conjugated chitosan-molecular beacon nanoparticles (CS-MB-OCT) can specifically bind to SSTR2 expressed by the lung cancer cells to achieve the goal of identification of lung cancer cells and imaging miR-155 *in vivo* and *in vitro*. Fluorescence imaging at different disease stages of lung cancer in Lox-Stop-lox K-ras G12D transgenic mice was performed, and could dynamically monitor the occurrence and development of lung cancer by different fluorescence intensity ranges. The current research, in turn, provides new idea, new method, and new technology for the early screening of lung cancer.

## Introduction

Lung cancer is one of the leading reasons for cancer-related mortalities worldwide (Wang et al., 2017). Due to lack of specific clinical manifestations in the early stage of lung cancer, most patients are diagnosed in the advanced stages. The early screening of lung cancer enables patients to obtain reasonable treatment at early stage and greatly reduce the cost of treatment and mortality. At present, the common methods for early screening include thoracic low-dose spiral computed tomography, protein array, biomarker, the organic compounds detected in the breath (Pan et al., 2017; Du et al., 2018; Rocco et al., 2018), and so on. But there are some limitations. So far, there is still lack of specific and sensitive molecular targets for early screening of lung cancer.

Studies have shown that microRNAs are widely involved in the occurrence, development, metastasis, recurrence of tumors, and emerged as a new cancer biomarker (Lu et al., 2017; Lee et al., 2016). Identification of relevant miRNAs in early-stage lung cancer is an important strategy for the early screening of lung cancer (Zhang et al., 2017). Current studies have shown that miR-155 was abnormally expressed in lung cancer, Lung cancer patients with high expression of miR-155 demonstrated poor prognosis and shorter survival time. The expression of miR-155 was also significantly increased in the serum of lung cancer patients, indicating it as a potential molecular marker for early screening and targeted therapy of lung cancer (Liu et al., 2017; Xue et al., 2016; Xie et al., 2015).

Molecular beacon (MB) technology is a very sensitive method used for detecting DNA, RNA, and microRNA (Kang et al., 2011; Zhang et al., 2017; Dong et al., 2016; Lee et al., 2016). Chitosan (CS) nanoparticles had been widely used to mediate gene transfection such as plasmids, siRNAs, microRNA, and demonstrated high safety and effectiveness (Geng et al., 2013; Raftery et al., 2013). In the previous studies, we have successfully used chitosan nanoparticles as miR-155 molecular beacon vehicles to detect miR-155 and imaging in the lung cancer cells (Zhu et al., 2014).

At present, the main challenge for cancer diagnosis and treatment is to enable anticancer drugs or imaging agents that specifically target tumor cells. The somatostatin receptors (SSTRs) are expressed in many tumor tissues and metastatic lesions, such as lung cancer, breast cancer, pancreatic cancer. Five subtypes of somatostatin receptors SSTRs, namely SSTR1-5, and SSTR2 expression are seen in most of these tumors (Shahbaz et al., 2015; Kharmate et al., 2013). Artificially synthesized somatostatin analog (SSTA) such as octreotide (OCT) could specifically bind to SSTR2 to achieve the purpose of targeting tumor cells with SSTR2 expression (Ju et al., 2018; Shen et al., 2017).

**Figure 1.**
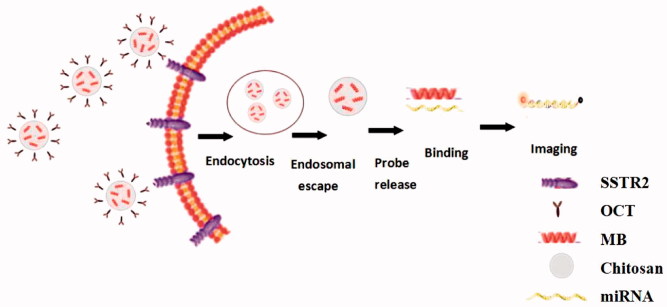
Schematic illustration of transfection of miR-155 MB into the cells via CS-MB-OCT nanoparticles for imaging of intracellular miRNA.

In Lox-Stop-lox (LSL) K-ras G12D transgenic mice model, lung adenocarcinoma can be induced by intranasal delivery of Cre adenovirus at adulthood, which thus dynamically displayed the processes from normal lung tissue to atypical hyperplasia, adenoma, carcinoma *in situ*, and adenocarcinoma (DuPage et al., 2009). It provides a good research model for the early diagnosis of lung cancer. In this study, we used lung cancer cells, subcutaneous xenograft model, and transgenic mice model as study subjects, combined the molecular beacons technology and nanotechnology, targeted OCT conjugated chitosan-molecular beacon (CS-MB) nanoparticles to synthesize CS-MB-OCT. OCT with its specificity to bind to SSTR2 on the surface of lung cancer cells, we achieved the goal of recognizing lung cancer cells by identification and imaging of miR-155 at both cellular and animal levels (Figure 1). This, in turn, provided the new idea, new method, and new technology for the early screening of lung cancer.

## Materials and methods

### Cell culture

Human lung adenocarcinoma cells A549 and prostate cancer cells PC-3 were purchased from ATCC, and human lung adenocarcinoma cells SPC-A1 cells were purchased from the Shanghai Institute of Cellular Biology. The cells were cultured in RPMI-1640 medium (Hyclone, USA) containing 10% fetal bovine serum (FBS, Invitrogen), and were placed in the incubator at 37 °C in 5% CO_2_.

### Animal model establishment

All *in vivo* experiments were approved by the Xinqiao Hospital Animal Care and Use Committee. Under sterile conditions, subcutaneous xenograft models were established by subcutaneous injection of 1 × 10^6^ A549, SPC-A1, and PC-3 cells into the right upper back of the mice (*n* = 6). Eight weeks after the transgenic mice were born, 5 × 10^9^ PFU Cre adenovirus（Hanbio, China） secreting the Cre enzyme was slowly instilled into the nasal cavity (*n* = 6). The mouse were sacrificed at 4 weeks, 6 weeks, 8 weeks, and 12 weeks after the adenovirus was instilled to establish different disease stages of lung adenocarcinoma models.

### SSTR2 expression detection

Immunofluorescence technology was used to detect the expression of SSTR2 in lung cancer cells and subcutaneous xenografts tissues. The cells were seeded in 24-well plates and were fixed by 4% paraformaldehyde(Boster, China), 1:200 primary antibodies for SSTR2 （abcam, UK）were added and then kept in the refrigerator at 4 °C for overnight incubation. The Alexa488-labeled (Alexa488:excitation/emission:490/520 nm) goat anti-rabbit secondary antibody (ZSGB-bio, China) with 1:200 dilutions was added, and then incubated at 37 °C for 30 minutes. The nuclei were then stained with 4,6-diamidino-2-phenylindole (DAPI) and the expression of SSTR2 was observed by confocal microscopy. When the size of the subcutaneous xenografts reached approximately 20 mm in diameter and 2500 mm^3^ in volumes, the tumors were removed and sliced into frozen sections. The same method was used to detect the expression of SSTR2 in subcutaneous xenograft tissues (*n* = 6). The transgenic mice were sacrificed at 4 weeks, 6 weeks, 8 weeks, and 12 weeks, respectively after the adenovirus was instilled. One side of the lung tissues of the transgenic mice at different disease stages were cut to prepare 4 mm paraffin sections after fixation with 4% formaldehyde. HE staining was then performed to observe the different pathological changes. Part of the subcutaneous xenografts and lung tissues from the other side were frozen in the liquid nitrogen for detecting miR-155. Immunohistochemistry was used to detect the expression of SSTR2 in lung tumor tissues at different disease stages (*n* = 6). The 1:200 SSTR2 primary antibodies were instilled and kept at 4 °C for overnight incubation. The biotin-labeled secondary antibody (ZSGB-bio, China) was added after washing with PBS and was incubated at 37 °C for 30 minutes. After washing three times with PBS for 3 min, horseradish peroxidase-labeled streptavidin was added. DAB staining was performed after washing with PBS, and then underwent counterstaining and sealing. Light microscopy was used to observe the SSTR2 expression.

### miR-155 expression detection

Real-time qRT-PCR was used to detect miR-155 expression in the subcutaneous xenografts and at different disease stages of lung tissues in transgenic mice. Part of the subcutaneous xenografts and lung tissues at different disease stages in the transgenic mice were taken out from liquid nitrogen with a weight of 0.1 g, respectively. RNAiso plus reagent (TaKaRa, Japan) was used to extract RNA. The U6 gene (Riobio, China) was chosen as the internal reference gene and miR-155 as target gene. The changes of miR-155 expression in xenografts and transgenic mice lung tissues were detected according to the manufacturer’s instructions of SYBR PCR Master Mix reagent kits (TaKaRa, Japan), (*n* = 6).

### Design and synthesis CS-MB-OCT

According to the previous research method, CS-miR-155 MB was synthesized in PBS (pH = 6.0) buffer system, where the molecular beacon (Sangon Company, China) weight was 2 μg and the chitosan nanoparticle (Guanghan Hengyu Company, China) was 14 μg. Chitosan nanoparticles and OCT (GL Biochem, China) molecules both contained amino and chemical cross-linker glutaraldehyde that combines the two. The methods are referenced in the literature and improved accordingly (Abdolmaleki et al., 2018; Gür et al., 2018; Gabriel et al., 2017; Sun et al., 2017). The CS-MB and OCT were simultaneously added in the 1% glutaraldehyde solution system. The weight ratio of OCT and CS was fixed at 4:7. After vortex oscillation and mixing for 60 s, and shaking on a 50 rpm shaker for 4 h, the solution was transferred to a microdialysis column and the cutoff molecular weight of the dialysis column was 50 kd. By dialyzing in PBS (pH = 6.0) solution for 4 h, the CS-MB-OCT was obtained. OCT-FITC (FITC excitation/emission:494/517 nm) was purchased from Shanghai GL biochem company, the same method was used to obtain the CS-MB-OCT-FITC for the purpose of identification and imaging of miR-155 *in vitro*.

### Characterizations of CS-MB-OCT

The 100 μlCS, CS-MB, and CS-MB-OCT solutions were added to 96-well plates (*n* = 3), respectively. The scan wavelength was set at 200–600 nm. PBS was made to zero. The Varioskan Flash (Thermo, USA) was used to detect the absorption spectra of the three materials. 1 ml CS-MB-OCT was added to the U-tube, where the temperature was set to 25 °C and Malvern Zetasizer Nano instrument (Malvern rn 3000HS, UK) was used to measure the average particle size. A drop of (CS MB-OCT), about 20 µl, was taken and instilled on the copper mesh to hold for 10 minutes and then dried by the filter paper. 2% phosphotungstic acid negative staining was performed for 1 minute. High-resolution TEM (Philips TECNAI 10, Holland) was performed to observe the CS MB-OCT morphology and photographing.

### Confocal microscopy and flow cytometry assay

The ability of CS-MB-OCT nanoparticles that target the SSTR2 on the cell membrane and detect the target miRNA was investigated. 1 × 10^4^ A549, SPC-A1, and PC-3 cells were seeded into the dishes and allowed to adhere. After 24 h incubation, they were washed three times for 3 min each with sterile PBS. 200 μl of CS-MB-OCT-FITC was added into the culture dishes, and then was supplemented with Opti-MEM I medium (Life Technologies, USA) to a total volume of 450 μl, making the final concentration of miR-155 MB and RS MB to 200 nM. The random sequence MB (RS MB) was used as a negative control. The cells were incubated in the CO_2_ at 37 °C for 60 minutes. 300 μl Hoechst 33342 (Beyotime, China) was added to stain the cell nucleus after washing three times for 3 min each with sterile PBS, and then the cells were incubated at 37 °C for 20 min. After washing with PBS, the cells were observed and photographed by confocal microscopy. After taking images, 1 ml cell lysis was added to fully lyse the cells. The liquid suspension was plated into 6-wells of the black 96-well plate with 100 μL (*n* = 6), Varioskan Flash was used to measure the Cy5(excitation/emission:649/670 nm) fluorescence intensity of the three cell lines. Using the same method, after transfection of miR-155 MB into A549, SPC-A1, and PC-3 cells with (CS MB-OCT), 1 ml 0.25% trypsin digestive solution was used to digest cells, and the transfection efficiency of CS MB-OCT was examined by flow cytometry (BD FACS Calibur, Becton, USA).

### Identification and imaging of miR-155 in vivo

The ability of CS-MB-OCT nanoparticles that target the SSTR2 on the cell membrane and detect target miRNA in vivo was further investigated. Depilatory cream was used for removing the black hair on the thorax for the purpose of detection of Cy5 fluorescence signal. After anesthetizing the mice with intraperitoneal injection of 4% chloral hydrate, 100 μl of CS-MB-OCT or CS-MB were injected into the tail vein. The CS-RS MB was used as negative control. The final concentration of the miR-155 MB or RS MB was 2 μM. After 2 hours, the xenografts nude mice or transgenic mice were put on the IVIS platform to detect fluorescence signals by IVIS spectrum imaging system (Caliper Life Sciences, USA). After imaging *in vivo*, the mice were sacrificed by cervical dislocation. The xenografts and lung tissues from the transgenic mice were removed and imaged again. After imaging, frozen sections were made using the xenografts or lung tissues. After paraformaldehyde fixation and DAPI staining of cell nucleus, the fluorescence signal intensity from frozen tissues was detected by confocal microscopy.

### Statistical analysis

An independent sample *t*-test was used for comparison between the two groups, and a one-way analysis of variance was used for statistical analysis among multiple groups. *p <* .05 was considered as statistically significant. Data were expressed as mean ± SD.

## Results and discussion

### Expression of SSTR2 and miR-155 in lung cancer cells, xenograft tissues and transgenic mice of different disease stages

Due to the differences in EPR (enhanced permeability and retention effect) between different tumors, active targeting strategies using nanomaterials often could achieve ideal results (Yuan et al., 2018; Badran et al., 2017). Hence, polypeptides or macromolecular polymers can be used to modify the surface of nanomaterials, combine the passive targeting of nanomaterials with the active targeting of polypeptides, thereby improving the targeting of tumor cells (Kim et al., 2012; Lapa et al., 2016). For example, anticancer experiments were conducted utilizing the octreotide-conjugated polymeric prodrug of bufalin for targeted delivery to breast cancer (Liu et al., 2016) and in a different study paclitaxel-octreotide conjugates were used as selective-targeted chemotherapeutic agents for treating non-small cell lung cancer (Sun et al., 2007).

**Figure 2.**
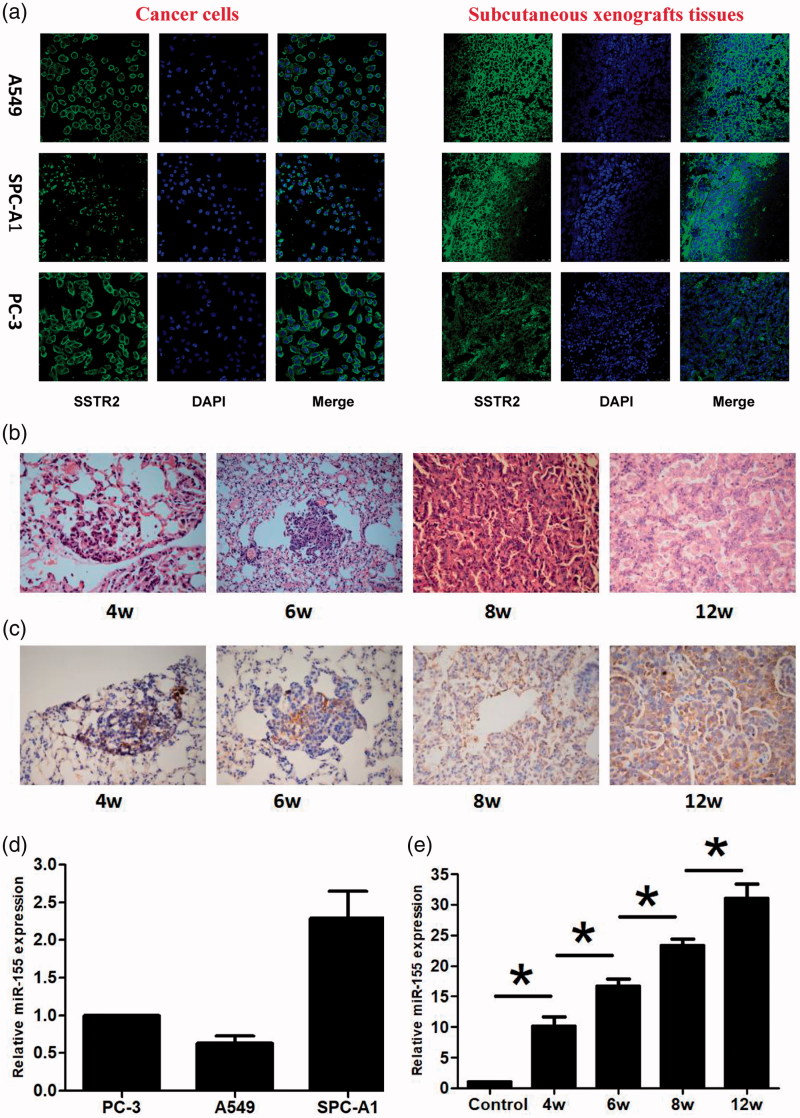
SSTR2 and miR-155 expression detection and animal model establishment. (a) SSTR2 expression by immunofluorescence. Scale bar = 50 μm. (b) HE staining at 4, 6, 8 and 12 weeks. (c) SSTR2 expression by immunohistochemistry (×400). (d) miR-155 expression in the subcutaneous xenografts (*n* = 6). (e) miR-155 expression in transgenic mice (*n* = 6). Data are presented as mean ± standard deviation (*p ＜ .05).

OCT is an artificially synthesized small-molecule of 8-peptide compound that binds to SSTR2 with high affinity. SSTR2 is expressed in lung cancer, breast cancer and other tumors (Walker et al., 2017; Mikołajczak et al., 2006). In this study, Confocal microscopy scanning revealed green fluorescence on A549, SPC-A1, and PC-3 cancer cells at both cellular and xenograft tissue levels. The location of expression was observed in the cell membrane and cytoplasm, indicating SSTR2 expression (Figure 2(a)). LSL K-ras G12D transgenic mice model provides a good research model for the early screening of lung cancer. HE staining found different pathological changes at 4, 6, 8 and 12 weeks after instillation of adenovirus, which included atypical hyperplasia, adenoma, carcinoma in situ, and adenocarcinoma in the transgenic mice lungs (Figure 2(b)), indicating the successful establishment of the model. Immunohistochemistry detection further demonstrated that SSTR2 was highly expressed in different stages of the diseased tissues (Figure 2(c)). Furthermore, it was available to target the tumor cells according to the characteristics of specific binding between OCT and SSTR2 to achieve the purpose of identification and imaging tumor cells.

MB technology has been extensively applied to detecting DNA, RNA, microRNA in living cells, rapidly analyzing gene mutation and biosensors, and so on. In our research, qRT-PCR assay showed that miR-155 was expressed in A549, SPC-A1 and PC-3 xenograft tissues and in different disease stages of transgenic mouse model (Figure 2(d)). In addition, the expression of miR-155 was increased with the disease progression of lung cancer. The differences of miR-155 expression were statistically significant among the five groups (Figure 2(e)), indicating that miR-155 expression was gradually increased during the occurrence and development of lung cancer, and can be used as a target for detection, tracing and early screening of lung cancer by MB technology. What is more, this technology could be further researched for future applications in the clinic.

### Design and synthesis of optimum CS-MB-OCT as a cancer diagnosis probe

**Figure 3.**
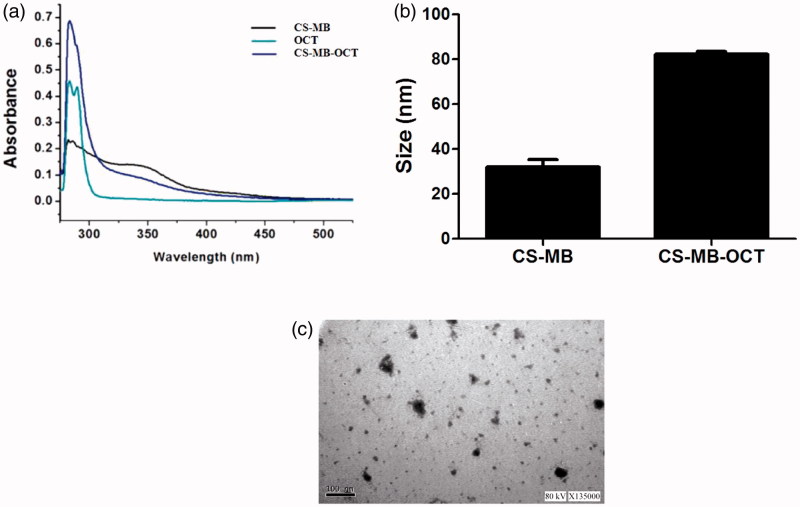
Physicochemical characteristics of CS-MB-OCT nanoparticles. (a) The CS-MB, OCT and CS-MB-OCT absorption spectrum. (b) The mean size of CS-MB and CS-MB-OCT (*n* = 3). The data were displayed as mean ± standard deviation. (c) TEM images of CS-MB-OCT. Scale bar =100 nm.

Chitosan nanoparticles have been widely used to mediate gene transfection of plasmids, siRNAs, microRNA, drug delivery and large quantities can be obtained from nature (Ganguly et al., 2015; Huang et al., 2017). The toxicity and immunogenicity were relatively low, and these can self-assemble into stable core-shell structures with RNA or DNA to protect RNA or DNA from degradation by serum enzymes. The chitosan nanoparticles delivery system ensures the efficiency of gene delivery and accurate expression (Sato et al., 2017). Based on the above studies of OCT and chitosan, OCT in this study was conjugated to the CS-MB, enabling the CS-MB to better exert the function of recognizing tumor cells. Chitosan nanoparticles and OCT molecular structures contained amino group. The chemical cross-linker glutaraldehyde can covalently bind with amino in the chitosan and OCT molecules, making OCT conjugated to chitosan (Yu et al., 2017). The black and blue-green lines indicated the absorption spectra of CS-MB and OCT, respectively. Both the absorption peaks were observed between 250–300 nm. The blue lines indicated CS-MB-OCT, which had the co-absorption spectrum of CS-MB and OCT, indicating successful connection between CS MB and OCT (Figure 3(a)). Nanoparticle size analysis and TME detection showed that the average particle size of CS-MB-OCT was about 80 nm, and CS-MB-OCT nanoparticles were relatively uniform and irregular spheroid particles (Figure 3(b,c)). The smaller nanoparticle size was suitable for cellular endocytosis and met the requirement of gene transfection.

### Fluorescence imaging and identifying of miR-155 in viable cells

**Figure 4.**
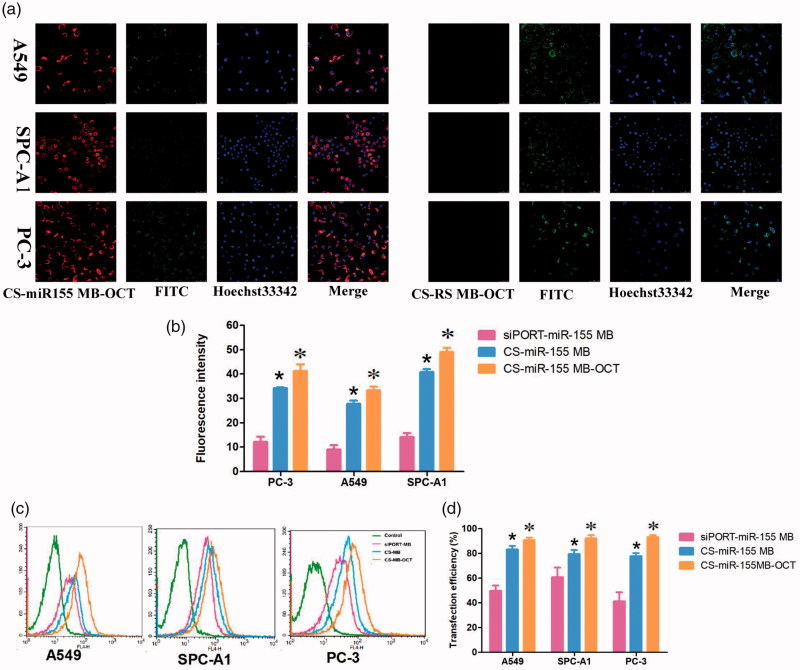
Fluorescence imaging and identification of miR-155 *in vitro*. (a) Confocal microscopy imaging. Scale bar = 50 μm. (b) Fluorescence intensity of miR-155 was measured after transfection with CS-miR155 MB-COT, CS-miR155 MB or siPORT-miR155 MB (*n* = 3). (c, d) Graphs by flow cytometry analysis and transfection efficiency of the three cell lines transfected with CS-miR155 MB-COT, siPORT-miR-155 MB and CS-miR-155 MB (*n* = 3). (*p ＜ .05, vs siPORT-miR-155 MB; *p ＜ .05 vs CS-miR-155 MB).

At the cellular level, *in vitro* experiments showed that in the presence of CS-miR-155 MB-OCT-FITC, strong red fluorescence signals were observed in all groups of cells, and most of which were located in cytoplasm and a few in the nuclei. Meanwhile, the green fluorescence emitted by the fluorescent dye FITC was also detected, which occurred due to the binding of OCT-FITC to the SSTR2 receptor on the cell membrane (Figure 4(a)). However, the CS-RS MB group showed no red fluorescence signal but only the green fluorescence was emitted by the fluorescent dye FITC. By combining the previous fluorescence intensity analysis (Figure S2) (Zhu et al., 2014). The fluorescence intensity of PC-3, A549, and SPC-A1 cells appeared to be stronger after miR-155 MB transfection in CS-MB-OCT group than that of CS-MB transfection group and siPORT liposome transfection group, indicating that OCT can target and bind to SSTR2. This, in turn, promotes more CS-MB-OCT nanoparticles that are endocytosed into the cells, making molecular beacons more likely to enter the cytoplasm and bind to intracellular miR-155. What is more, the tendency of fluorescence intensity in the CS MB-OCT transfection group was consistent with the tendency of miR-155 expression in qRT-PCR (Figure 4(b)). Flow cytometry examination showed that the transfection efficiency was observed to be the highest in the CS-miR-155 MB-OCT group (Figure S3, Figure 4 (c,d)), further demonstrating that OCT targeted lung cancer cells effectively with high transfection efficiency. All indicated that the OCT exerts a targeting effect and specifically binds to SSTR2, promoting more CS-MB-OCT to be endocytosed into the cells, and made the MB more likely to enter the cytoplasm and bind to the intracellular miR-155.

### Fluorescence imaging and identification of miR-155 in subcutaneous xenografts of nude mice

At the living animal level, IVIS spectrum imaging system after injection of CS-MB-OCT or CS-MB nanoparticles into the tail vein detected the fluorescence signals from the xenografts and indicated the distribution of fluorescence signals were consistent with tumor size (Figure 5(a)). In addition, the fluorescence signals were stronger in the CS-MB-OCT group. After the tumor tissues were removed, the imaging examination showed that the fluorescence signals were originated from the tumor tissues (Figure 5(b)). By analyzing the intensity of fluorescence signals generated by the xenografts using the Spectrum image 4.0 software, the fluorescence intensity remained stronger in the CS-MB-OCT group compared to those of the CS-MB group (Figure 5(c)). No significant fluorescence signals were detected in the CS-RSMB-OCT negative control group. After the frozen sections of the tumor tissues were made again, the confocal microscopy examination showed that the CS-MB-OCT group had the strongest fluorescence signals, which were mainly localized in the cytoplasm (Figure 5(d)). This indicated that CS-MB-OCT nanoparticles can target and bind to the SSTR2 receptors on the cell membrane, promoting more CS-MB-OCT nanoparticles to enter the cell. This subsequently allows more molecular beacons to enter the cytoplasm and bind to intracellular miR-155, resulting in stronger fluorescence signals.

Figure 5.Fluorescence imaging and identification of miR-155 *in vivo*. (a) IVIS spectrum imaging system of subcutaneous xenografts after injection of CS-MB-OCT or CS-MB nanoparticles. (b) IVIS spectrum imaging of the xenograft after removal (c) Fluorescence intensity was measured after injection (*n* = 6).（*p ＜ .05, vs *CS-RS MB*；*p ＜ .05 vs CS-miR-155 MB）(d) Confocal microscopy imaging of the tumor tissues after transfection with CS-miR155 MB-OCT, CS-miR155 MB or CS-RS MB. RS MB was used as a negative control. Cell nuclei were stained by DAPI (blue). Scale bar = 25 μm.

**Figure:**
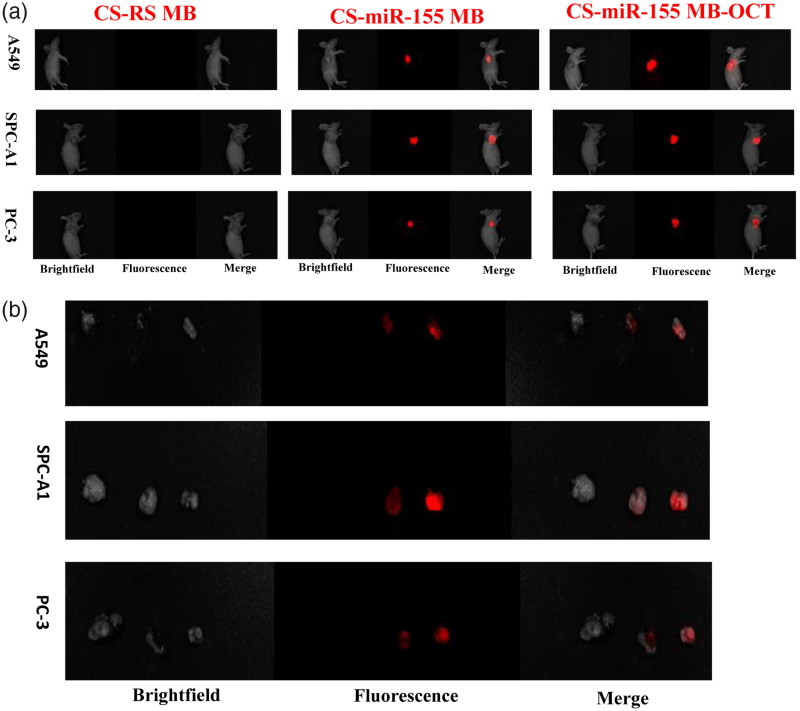

**Figure:**
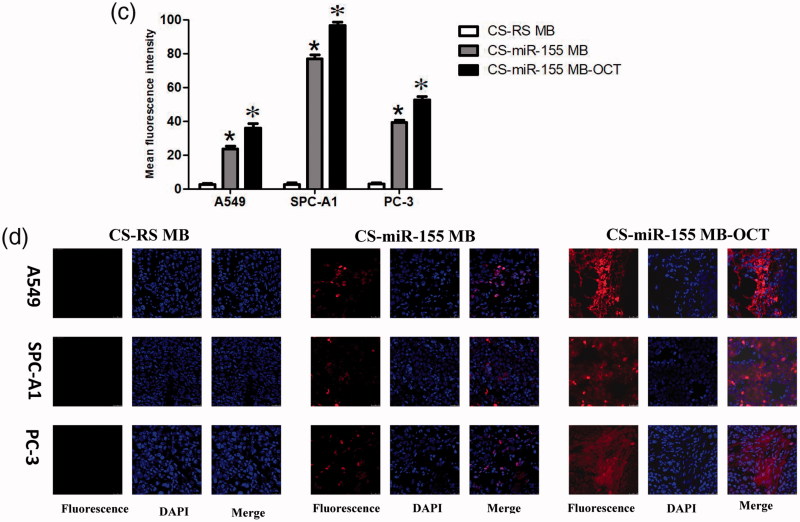

### Fluorescence imaging and identification of miR-155 in transgenic mice models

**Figure 6.**
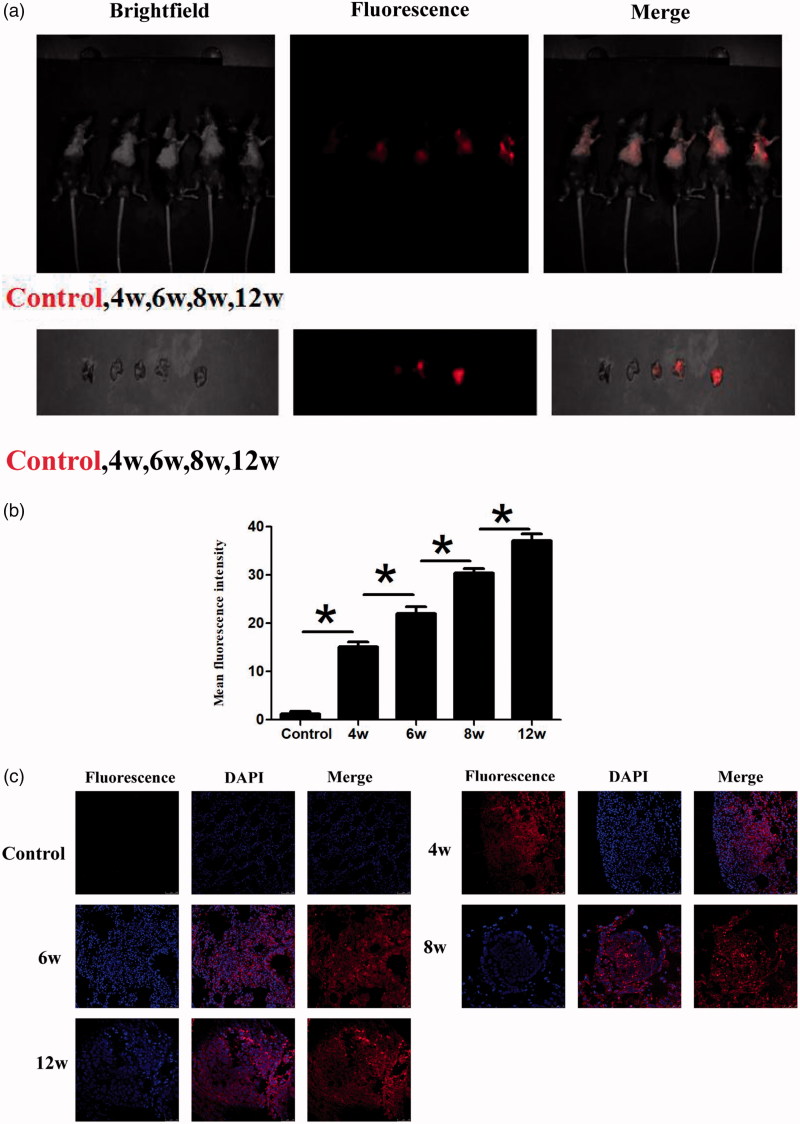
Fluorescence imaging and identification of miR-155 of transgenic mice at different disease stages. (a) IVIS spectrum imaging system imaging after injection of CS-MB-OCT into the tail vein. (b) IVIS spectrum imaging system of the lungs after removal. (c) Fluorescence intensity was measured after injection (*n* = 6, *p < .05). (d) Confocal microscopy of different pathological changes after transfection with CS-MB-OCT. Scale bar = 25–100 μm.

After we injected CS-MB-OCT nanoparticles into the established transgenic mice models from the tail vein, strong or weak fluorescence signals were detected in the lungs of transgenic mice, and the fluorescence signals were gradually increased with the progression of the disease. However, the fluorescence signals were not detected in the control group mice without intranasal inhalation of the adenovirus (Figure 6(a)). After the lungs were removed, the fluorescence signals were originated from the lungs and were gradually increased with the progression of the disease (Figure 6(b)). The mean fluorescence intensities were about 15.13 ± 1.00, 22.00 ± 1.43, 30.39 ± 0.96, 37.08 ± 1.46 at the atypical hyperplasia, adenoma, carcinoma in situ, adenocarcinoma of different disease stage in vivo, respectively (Figure 6(c)). After frozen sections of the lung tissues were made, confocal microscopy revealed that the fluorescence signal was derived from the tumor cells (Figure 6(d)). The OCT-targeted mediation increased the endocytosis of nanoparticles by the cells, so that the molecular beacons were more efficiently accumulated in the tumor cells and entered the cells to imaging the microRNAs of the tumor cells, thus generating strong fluorescence signals. What is more, as the miR-155 expression went up, the fluorescence signals were also increased, thus achieving the purpose of early screening of tumors by identification and imaging of miR-155 expression in the tumor tissues. By dynamically monitoring the occurrence and development of lung cancer according to the differences in fluorescence intensity ranges, a new method and new idea for the early screening of lung cancer was provided.

## Conclusions

In summary, the CS-MB-OCT nanoparticle probes were synthesized. Both *in vivo* and *in vitro* experiments showed that the synthesized CS-MB-OCT specifically binds to SSTR2 on the surface of tumor cells to exert its targeting effect, to recognize and image miR-155 expressed in the lung cancer cells, lung xenografts of nude mice and transgenic mice of different disease stages. The dynamic monitoring of the occurrence and development of lung cancer by different fluorescence intensity provided new ideas and new experimental evidences for the early screening of lung cancer. The same method can also be used for early screening of other organ tumors.

## Supplementary Material

Supplementary_Material_-___.pdf
