# Supplementary material for: Identification and imaging of miR-155 in the early screening of lung cancer by targeted delivery of octreotide-conjugated chitosan-molecular beacon nanoparticles
Source: Drug Deliv. 2019 Jan 9;25(1):1974–83. doi: 10.1080/10717544.2018.1516003 (PMC6327580; doi:10.1080/10717544.2018.1516003)
Supplement: Supplementary_Material_-___.pdf [file IDRD_A_1516003_SM7380.pdf]

Supplementary data associated with this article Fig S1-3 could been found in the online version at doi:10.3390/molecules190914710. In articl: Molecules 2014,19,14710-14722;

Figure S1. Physicochemical characteristics of CS-MB nanoparticals complexes. TEM images of CS and CS-MB. Scale bar = 100 nm.

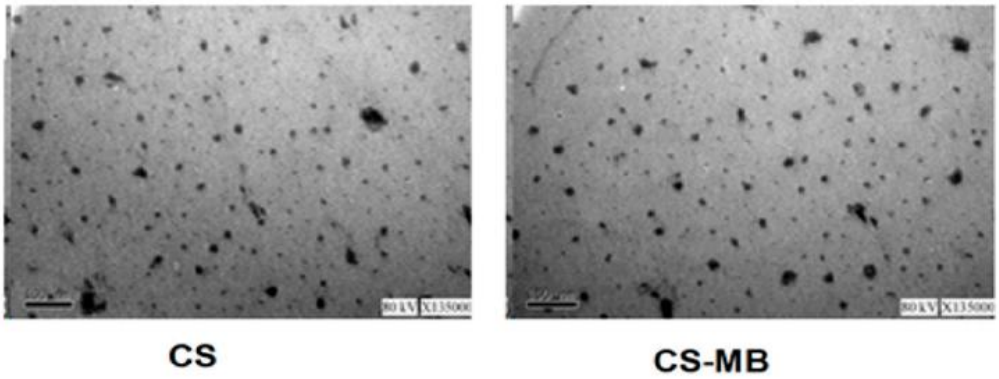

Figure S2. Fluorescence intensity of Cy5 was measured after incubation with CS-miR155 MB or siPORT-miR155 MB. * $p < 0.05$.

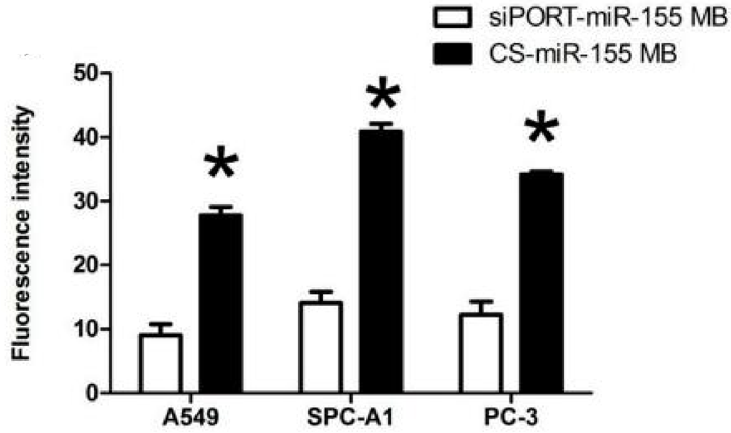

Figure S3. Graphs by flow cytometry analysis and transfection efficiency of the three cell lines transfected with siPORT-miR-155 MB and CS-miR-155 MB (n = 3). * $p < 0.05$.

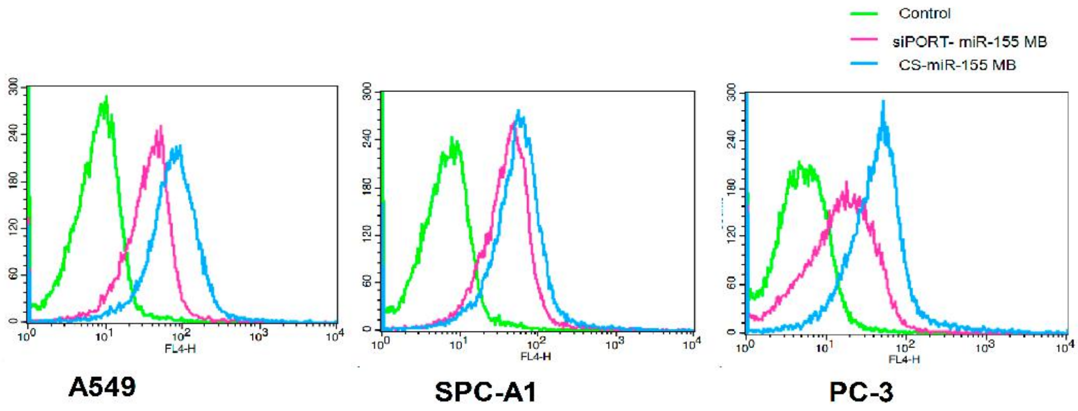

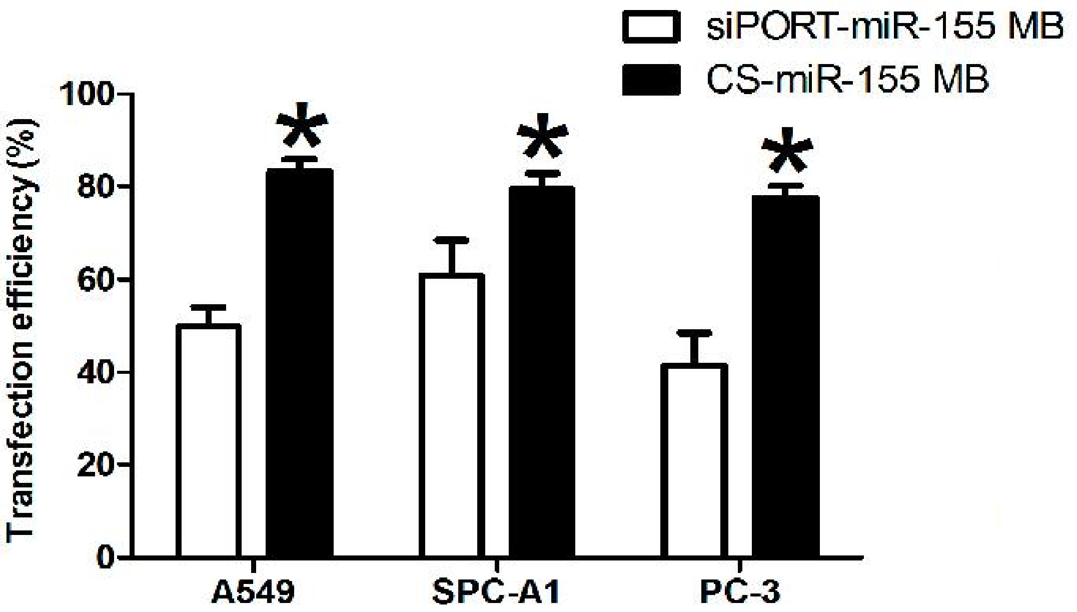
siPORT-miR-155 MB
CS-miR-155 MB
Transfection efficiency (%)
100
80
60
40
20
0
*
*
*
A549
SPC-A1
PC-3
